# Physical Routes to Primitive Cells: An Experimental Model Based on the Spontaneous Entrapment of Enzymes inside Micrometer-Sized Liposomes

**DOI:** 10.3390/life5010969

**Published:** 2015-03-18

**Authors:** Erica D’Aguanno, Emiliano Altamura, Fabio Mavelli, Alfred Fahr, Pasquale Stano, Pier Luigi Luisi

**Affiliations:** 1 Science Department, Roma Tre University, Viale G. Marconi 446, I-00146 Rome, Italy; E-Mails: erica_daguanno@yahoo.it (E.D.); emiliano.altamura@uniba.it (E.A.); 2 Institut für Pharmazie, Friedrich-Schiller-Universität Jena, Lessingstraße 8, D-07743 Jena, Germany; E-Mail: Alfred.Fahr@uni-jena.de; 3 Chemistry Department, University of Bari, Via E. Orabona 4, I-70125 Bari, Italy; E-Mail: mavelli@uniba.it

**Keywords:** carbonic anhydrase, confocal microscopy, crowding, spontaneous concentration, enzymes, lipid vesicles (liposomes), primitive cells, proteinase K, self-organization

## Abstract

How did primitive living cells originate? The formation of early cells, which were probably solute-filled vesicles capable of performing a rudimentary metabolism (and possibly self-reproduction), is still one of the big unsolved questions in origin of life. We have recently used lipid vesicles (liposomes) as primitive cell models, aiming at the study of the physical mechanisms for macromolecules encapsulation. We have reported that proteins and ribosomes can be encapsulated very efficiently, against statistical expectations, inside a small number of liposomes. Moreover the transcription-translation mixture, which realistically mimics a sort of minimal metabolic network, can be functionally reconstituted in liposomes owing to a self-concentration mechanism. Here we firstly summarize the recent advancements in this research line, highlighting how these results open a new vista on the phenomena that could have been important for the formation of functional primitive cells. Then, we present new evidences on the non-random entrapment of macromolecules (proteins, dextrans) in phospholipid vesicle, and in particular we show how enzymatic reactions can be accelerated because of the enhancement of their concentration inside liposomes.

## 1. Introduction

It is accepted that life on Earth originated from the inanimate matter through a very long and slow series of steps which brought about the transformation from small molecular precursors to primitive cells, and from the latter to the last universal common ancestor (LUCA), from which biodiversity was then generated. The first part of this narrative is supported by a remarkable series of prebiotic chemistry experiments (reviewed in [1]), whereas the nature of the LUCA can benefit—up to a certain extent—of reconstruction of the roots of the evolutionary trees by molecular phylogenetics and bioinformatics. The central part of the story, namely, on how cells have been originated by separated molecules and on the origin of those functional molecules, is largely unknown and it is also interrupted by several conceptual gaps. Unanswered questions about the synthesis of sufficiently long macromolecules, about their specific sequences and functions, or about the origin of genetic code, or about the emergence of early self-replicating entities hinder the full understanding of cellular origin. Among these questions, one of the most important refers to the origin of primitive cellular structures that preceded both in time and complexity the early full-fledged biological cells.

We do not know what was the structure of primitive cells—which were probably not fully autonomous (“limping” cells [2,3]). Possibly they were lipid vesicle containing a sort of rudimentary metabolic and genetic systems.

If proteins and nucleic acids came first, then how were all macromolecules entrapped in a single compartment? If, on the other hand, functional macromolecules originated from inside the compartment that would mean that we then have to conceive semi-permeable, sophisticated membranes in prebiotic times, which does not appear plausible.

In recent years, stimulated by our original observations on protein encapsulation inside fatty acid vesicles [4,5] and by the reconstitution studies based on protein synthesis inside phospholipid vesicles [6,7,8,9], we started a direct investigation of solute entrapment during liposome formation, with specific attention to macromolecular solutes. Our results, which have been recently published [10,11,12,13,14,15], possibly offer a partial solution to the question about the origin of cellular structures, because they show that proteins and ribosomes can be encapsulated with high efficiency inside liposomes (actually, inside a small number of liposomes in a population).

In this article we would like to firstly review the main results of previous investigations, including a short historical development of the field, then move to new data on the encapsulation of enzymes and other macromolecules in lipid vesicles. These new experiments were designed and realized specifically to show the spontaneous formation of enzyme-rich vesicles—here considered as primitive cell models—with an internal “metabolic” activity, when compared with the same non-confined system (the reaction in bulk). This provides a possible explanation to the origin of functional cells and at the same time opens a new vista on the principles of the entrapment of solute in vesicles (which might also be relevant for biotechnological applications).

## 2. The Minimal Cell and a Review of Previous Results on Macromolecules Entrapment in Liposomes (Part of the Discussion Derives from [13])

### 2.1. The Minimal Cell

Let us go back to the question, what kind of primitive cells emerged from prebiotic chemistry and that later brought to LU*C*A. The two possibilities—which are typically discussed—are protein-first and RNA-first scenarios. As commented in previous papers [3,13], both present advantages and disadvantages for a narrative of origin of life, mainly because these two classes of biopolymers excel in catalysis or in replication, and both are actually needed for constructing a living cell. The discovery of ribozymes [16,17] has greatly prompted a vision where RNA molecules alone were sufficient to achieve the needed cellular (and pre-cellular) functionality [18], and a RNA-protocell model has also been proposed [19,20], but several open questions on the origin of RNA world and on its evolution toward the LUCA’s RNA/protein/DNA world, with no experimental and theoretical answers, still remain.

It is quite plausible to suppose, however, that a sort of simplified bounded molecular system existed before the origin of the first full-fledged cell, and that such system contained the minimal and sufficient number of molecular components to be defined alive, or at least to display some of the most relevant features of living cells, such as compartmentalized reactions, ribosomal protein biosynthesis, the capacity of self-maintenance, and possibly self-reproduction.

**Figure 1:**
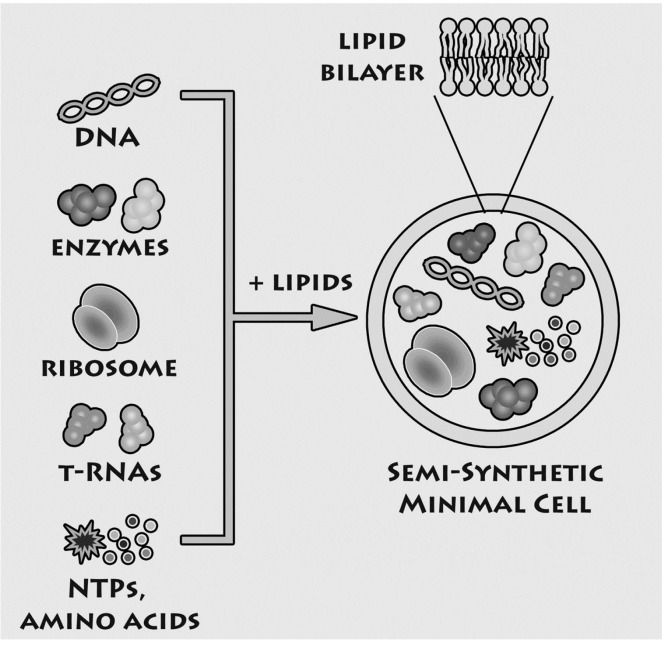
Experimental approach for constructing semi-synthetic minimal cell (reproduced from [23] with the permission of Elsevier).

In the last years, we have been concerned with experimental studies on the “minimal cell” [3], focusing on the simplest and most ancient possible structure of biological cells. Our approach is shown in Figure 1: we incorporate extant genes and enzymes inside lipid vesicles—which function as model of cell membrane. In particular, the term “semi-synthetic minimal cells” has been used to describe minimal cells models that can be realized in the laboratory. This approach has multifold advantages. Firstly, minimal cells can be built in the laboratory since all components are available. Therefore, minimal cells belong to the realm of laboratory approaches. Secondly, this “synthetic” approach [3,21,22] allows testing hypothesis about the minimal complexity required for cellular life. In principle, in fact, it is possible to reconstruct and study the desired functions in a fully artificial system that mimics the cellular structures that existed billions of years ago. Thirdly, physical effects, not only (bio)chemical ones, can be investigated, such as the entrapment of solutes—this will be the main topic of this article. Finally, the methods and strategies applied for constructing minimal cells in origin of life scenario can be exported to other fields, such as synthetic biology, biotechnology, nanomedicine.

**Figure 2:**
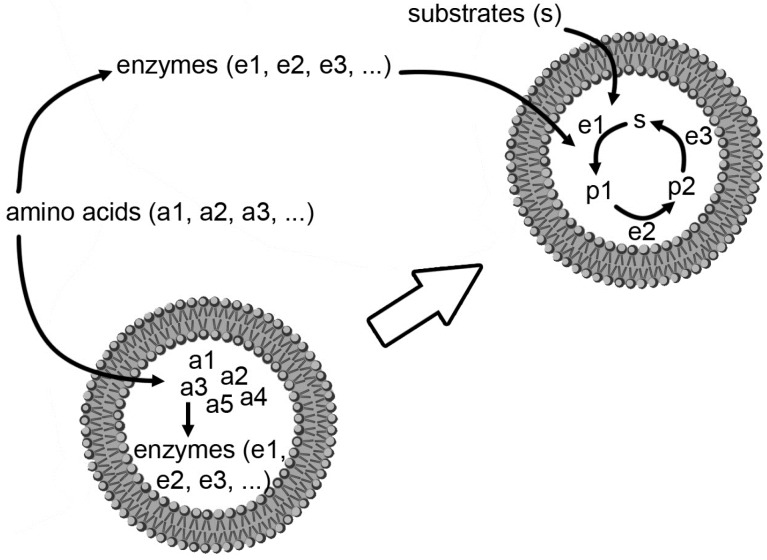
Two alternative (and perhaps competitive) hypothetical mechanisms for the formation of the first protocells, whereby the first proteins (and enzymes) were constructed inside a compartment (bottom), or first outside, then incorporated inside (top). Redrawn, with minor modifications, from [13].

How did *primitive* minimal cells originate? Even if these structures have, by definition, a minimal genetic/metabolic complexity, it is evident that they must contain hundreds of components, just to count the macromolecular (function-bearing) ones. Here two possibilities can be discussed (Figure 2). The first one is that the biochemical network developed firstly in the environment, and later become encapsulated inside lipid vesicles; the second one is that the network was born already within compartments, starting from simpler molecules. Both appear difficult. The first one because it is hundreds of macromolecules and small molecules should be encapsulated within the same lipid vesicle in order to have a functional cell; the second because during the (very long) process of network development, building blocks should enter the compartment, byproducts should leave it, and permeability should be somehow controlled in order to have such a sophisticated “bioreactor” that function correctly. Can experiments on *semi-synthetic* minimal cells—those that can be constructed in the laboratory—help to clarify, at least partially, such question?

### 2.2. Transcription-Translation Reactions inside Liposomes

**Figure 3:**
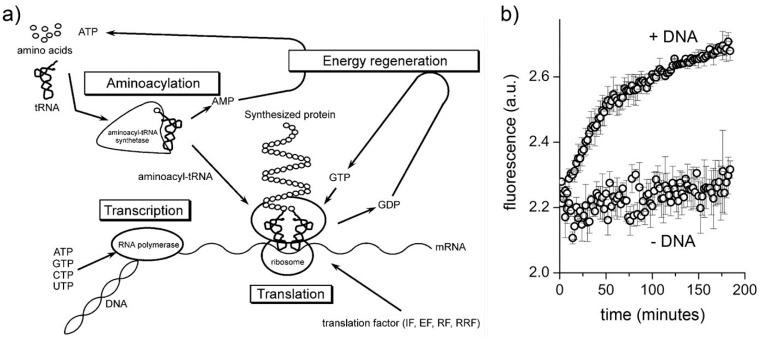
Protein synthesis inside conventional liposomes. (**a**) Components and functions of the PURE systems (reproduced from [27] with the permission of Elsevier); (**b**) fluorescence *versus* time profiles of eGFP producing vesicles (+DNA) and negative control (−DNA) (reproduced from [9] with the permission of Wiley).

Transcription-translation (TX-TL) network is the core set of reactions in minimal cells. The genes that encode such network constitute about 60% of the “minimal genome” [24,25], and TX-TL reactions can be carried out *in vitro* by using cell extracts (for example, from *Escherichia coli*) or reconstituted systems. For these reasons, the construction of semi-synthetic minimal cell encapsulating TX-TL mixtures is a quite advantageous way to mimic primitive cells of minimal yet not negligible complexity. The PURE system (Figure 3A) is a reconstituted TX-TL kit composed by the minimal number of components (about 80 macromolecules, two dozen small molecules, organic buffer, and inorganic salts) required for synthesizing a protein starting from DNA [26,27].

The PURE system and cell extracts have been used to produce functional proteins inside lipid vesicles (results reviewed in [21]). Starting from the aqueous solution of the PURE system, lipid vesicles are formed *in situ*, for example by swelling phospholipid films or by adding lipids as ethanol solution. In these conditions, lipid vesicles form spontaneously and it happens that they mechanically entrap the macromolecular components of the TX-TL kit. In order to produce a protein, all PURE system components must be necessarily present in the same lipid vesicle. Experimental results obtained with conventional vesicles of diameter < 300 nm [9] show that this is indeed the case (Figure 3B), even if, at first sight, such multimolecular co-entrapment appears to be statistically implausible [2].

Let us see the reasons. In order to make a quantitative estimate, it is possible to calculate the probability of simultaneous entrapment of all PURE system macromolecules inside lipid vesicles of a certain diameter according to standard theory. In particular, in agreement with the following null hypotheses (*H*_0_): the entrapment can be modeled as a random sampling event, and the average number of solute molecules found in a vesicle of volume *V* is simply μ = *N*_A_ *C*_bulk_ *V*, where *N*_A_ is the Avogadro’s number and *C*_bulk_ is the bulk solute concentration. If this is true, it also follows that the probability of finding *n* molecules in a vesicle (when μ are expected) is given by the Poisson statistics, *i.e.*, *p*(*n*) = *e*^−μ^ μ*^n^*/*n*!, and that the co-entrapment probability for *k* molecules is the product of the *k* individual entrapment probabilities [9]. As intuitively expected, the co-entrapment of one (or several copies) of each PURE system component is highly improbable inside small vesicles, and the probability values are of the order of 10^−26^. In other words, the results presented in [9] could be understood only by rejecting *H*_0_. In particular, calculations have shown that experimental data could be explained by supposing a spontaneous concentration of PURE system components (*i.e.*, *C*_vesicles_ > *C*_bulk_).

### 2.3. The Entrapment of Ferritin inside Liposomes and Other Recent Results

**Figure 4:**
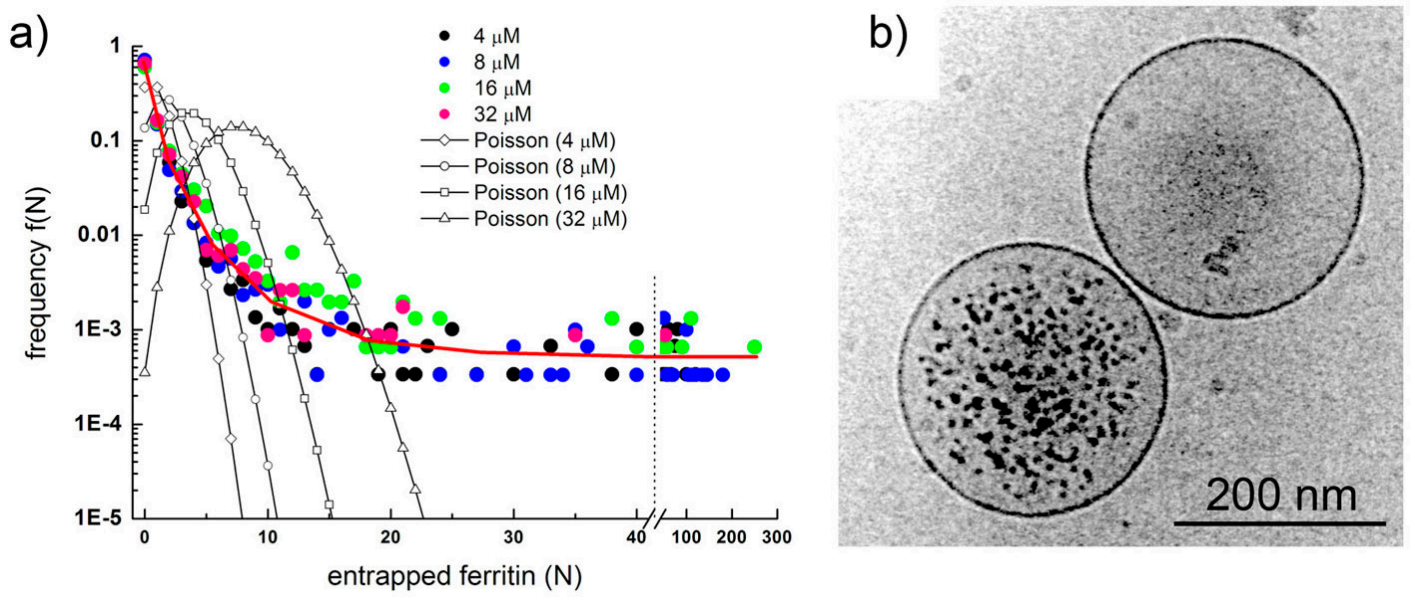
(**a**) Ferritin occupancy distribution as determined by analyzing about 7700 individual vesicles imaged by cryo-TEM. Experimental points shown as colored circles, theoretical expectations as empty circles connected by a line; (**b**) Two conventional POPC vesicles: one is super-filled with ferritin (encapsulating much more ferritin molecules than expected), the other does not contain any ferritin molecule (also against expectations). Images reproduced from [10] with the permission of Wiley.

Intrigued by these unexpected conclusions, we then started a direct investigation on solute encapsulation inside spontaneously formed lipid vesicles. We recalled our previous work on the use of the protein ferritin as a marker for the vesicle lumen [4,5]. Individual ferritin molecules contain high amount of iron in form of hydrous ferric oxide phosphate, and it is widely used in electronmicroscopy. Since the number of ferritin molecules inside vesicles can be directly counted in images obtained by cryo-transmission electronmicroscopy (cryo-TEM), and the vesicle size is measurable as well, it is possible to measure the solute occupancy distribution *f*(*n*) in vesicle populations and compare it with the expected Poisson distribution, *p*(*n*). As shown in Figure 4A, the observed distribution is quite different than the theoretical one. The distribution is not bell-shaped, and—at high *n*—the measured distribution has a long “tail” of values that are significantly higher than the vanishing small values of the Poisson curve. The experimentally determined distribution strongly resemble a power law, *i.e.*, *f*(*n*) ~ 1/*n*^a^ (a > 0), and Figure 4B summarizes in one micrograph the main message behind this study: as a result of spontaneous vesicle formation and solute encapsulation processes, empty and filled vesicles coexist in the same sample, and whereas it is evident that most of the vesicles contain a low number of ferritin molecules, or are empty, a minority of them (<1%) contains, against expectations, a very high number of ferritin molecules in non-aggregate state. The existence of these “super-filled” vesicles is considered almost impossible according to Poisson statistics, whereas is correctly predicted by the power law. Moreover, in the case of very small vesicles, exceptionally high intravesicle ferritin concentrations have been observed (up to about 300 μM), roughly corresponding to crowding concentrations in biological cells.

Experiments were repeated with ribosomes [11] and with peptidyl-RNA complexes [12], obtaining similar results. The scenario that is going to be disclosed is the following. When lipid vesicles, especially sub-micrometer ones, are formed in an aqueous phase containing macromolecular solutes, vesicle formation mechanisms and solute entrapment mechanisms bring about the formation of super-filled vesicles, as if solutes are sucked in, irrespective of the expected tendency of spreading in the largest possible volume. This happens, however, only for few special vesicles, probably those experiencing particular environmental local conditions that permit the onset of such a peculiar mechanism of solute encapsulation/vesicle formation. In absence of more detailed mechanistic information, we have made the hypothesis that such super-entrapment is based on the perturbation of the vesicle formation mechanism (*i.e.*, a kinetic effect; slowing down the closure of open lipid bilayers [28] due to solute-water-membrane interactions), whereas the driving force for the accumulation of molecules (*i.e.*, a thermodynamic effect) could be the cooperative release of bound water, as happens in the well-know hydrophobic effect.

Although more experimental and theoretical studies are required in order to verify this hypothetical mechanism (coarse-grained simulations seems to deny it [29,30]), its implications are clear: the formation of lipid vesicles has the potentiality of concentrating substances in their lumen. If this mechanism can be translated to origin of life scenario it means that when primitive cells were formed by self-assembly of membrane-forming compounds (very probably a mixture of amphiphiles, see [31]), the solutes present in the environment, and in particular macromolecular ones, could have been accumulated inside and this would have been the major factor for the origin of cellular metabolism. Very recently we have directly assayed this scenario by forming lipid vesicles in diluted (and therefore unreactive) PURE system. Green fluorescent protein synthesis was observed only inside (few) vesicles, suggesting that also in the case of multimolecular mixture a power law distribution ruled the solute encapsulation ([14]; Mavelli and Stano, manuscript in preparation). Together with cyro-electronmicroscopy observations of PURE system-filled vesicles [11], this could explain our original observation of protein synthesis inside small conventional liposomes [9] in terms of simultaneous concentration of several macromolecules.

## 3. New Results on the Encapsulation of Proteins and Dextrans in Vesicles

Having summarized the most relevant results on the investigations of macromolecular entrapment inside lipid vesicles, let us illustrate the motivations behind the approach presented here, and show new results.

Key evidences about the accumulation of macromolecular solutes inside liposomes have been achieved via cryo-TEM by using large unilamellar vesicles (typical diameter: 100–400 nm) and solutes that can be visualized by this technique (ferritin, ribosomes, peptidyl-RNA complexes). With this approach it is possible to visualize vesicles individually, to directly see their shape and lamellarity, and count individual molecules. However, there are also two limitations, namely the impossibility of following in real time biochemical reactions, and the quite narrow choice of solutes that can be visualized. For these reasons, a more versatile methodology would be helpful to extend the solute entrapment studies. Three possibilities are: confocal fluorescence microscopy, infrared microscopy [32], and flow cytometry [33,34].

We decided to extend our investigations by using confocal fluorescence microscopy. Fluorescence microscopy is particularly suitable for large vesicles (diameter > 0.4–0.5 μm), and can be used to assess the presence of solutes whose concentration can be measured, in direct or indirect way by a fluorescence signal. Fluorescently-labeled macromolecules or fluorescence-based enzymatic reactions are suitable for these purposes. We aim at studying the encapsulation of macromolecules like proteins, polysaccharides, and nucleic acids (D’Aguanno, manuscript in preparation). Both non-reacting and reacting systems will be presented here. These experiments will ultimately show that the formation of lipid vesicles might act as a sort of “attractors” for molecules present at low concentration in the environment. Such molecules, once encapsulated (and concentrated) inside vesicles, might overcome a concentration threshold that might trigger chemical reactions.

### 3.1. Liposome Preparation

In order to study the entrapment (encapsulation) of solutes in spontaneously formed vesicles we have chosen vesicle preparation methods that model as better as possible self-organization pathways, with minimal guidance by the operator. As lipids, we have employed the well-known 1-palmitoyl-2-oleoyl-*sn*-glycero-3-phosphatidylcholine (POPC), alone or in the presence of sodium oleate (20–50 mol%). Note that a similar study based on pure fatty acid vesicles—which are more realistic models of primitive cells—is currently in progress in our laboratory, and it will be published in a dedicated article (D’Aguanno, manuscript in preparation). We are aware of the fact that pure POPC vesicles probably do not represent the best primitive compartments model—although it has been, and is, used frequently, either because POPC is a phospholipid (whereas primitive lipids were simpler molecules, like fatty acids or isoprenoids), either because a *mixture* of simpler lipids, heterogeneous in terms of chain length and head group, are more plausible as components of early membranes. We will come back to this point in the concluding remarks.

We have optimized the preparation methods in order to obtain vesicles in the micrometer range (95% of vesicle diameters lies in the 0.83–2.5 μm range), so that their visualization by a common confocal microscope is non-ambiguous. The present study therefore differs from previous ones based on cryo-TEM (vesicle diameter < 0.4 μm) because it assays the encapsulation within larger vesicles. It also differs from a published study based on confocal microscopy [35] because the latter was focused on encapsulation in larger (giant) vesicles (vesicle diameter: 10–20 μm).

Method 1 (M1): *hydration of lipid film* [36]. This traditional vesicle preparation method simply consists in hydrating, with an aqueous solution, a previously dried lipid film. Generally the film is obtained starting from a solution of lipids in chloroform or similar solvent. In this study, for the matter of convenience (hydration with small volumes), we have deposited the lipid film over 2 mm glass beads, similarly to a previous report [37].

Method 2 (M2): *hydration of freeze-dried lipid vesicles* [38,39]. This method is similar to the simple film hydration, but instead of using a lipid film, 400 nm (or 800 nm) extruded and then freeze-dried lipid vesicles are used.

Method 3 (M3): *ethanol injection method* [40]. Lipid vesicles can be prepared by injecting a concentrated lipid solution in ethanol (or other water-soluble alcohols) into an aqueous solution. The size and morphology of resulting vesicles depend mainly from the type of lipids and from the concentration of lipids in the stock alcoholic solution [41,42]. In particular, large vesicles can be obtained when stock solutions of high concentration (100 mM) are used.

**Figure 5:**
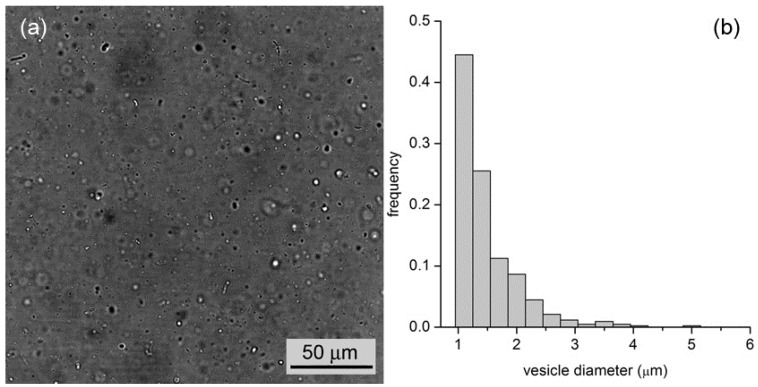
(**a**) Bright-field microscopy image of a vesicle sample as obtained by the film hydration method (M1); (**b**) Vesicle size distribution with an average diameter of 1.3 μm.

By all methods, we obtained vesicles in the micrometer range. In the case of M1, for example, vesicles with an average diameter of 1.3 μm (standard deviation ±0.6 μm, *n* = 427) and a wide size distribution (up to 5–6 μm) have been observed (Figure 5). In the case of M2 and M3, we obtained, respectively, fluorescent vesicles with diameters of 1.2 ± 0.3 μm (*n* = 48) and 1.4 ± 0.5 μm (*n* = 55).

### 3.2. Entrapment of Proteins and Dextrans

Next, we have investigated the entrapment of solutes inside micrometer-sized vesicles prepared by the abovementioned three methods. We have followed a simple and straightforward procedure, namely preparing a population of lipid vesicles in an aqueous solution that contain a solute of interest at a certain concentration (typically between 0.1 and 5 μM). Then we simply analyzed the so-obtained samples by confocal microscopy (all tested solutes were fluorescent). If the solute molecules, once entrapped, have the same concentration as the bulk solution, liposomes will not be distinguished from the background. If, on the other hand, more solute molecules are entrapped inside liposomes, these will appear more fluorescent than the background.

Preliminary calibration experiment served (*i*) to build a calibration line for converting fluorescence to concentration (this was done for different instrumental settings, including gain and offset); and (*ii*) to ensure that the fluorescence values in the accessible range scaled linearly with fluorochrome concentrations, meaning that the assayed concentration range did not suffer of self-quenching and/or inner filter effects.

We have employed common commercially available fluorescent proteins and dextrans with different molecular weights, in particular: dextran conjugated with rhodamine (dextran-RITC, *ca.* 10 kDa), phycoehrytrhin (PE, *ca.* 240 kDa), bovine serum albumine conjugated with fluorescein (BSA-FITC, *ca.* 66 kDa), allophycocyanin (APC, *ca.* 104 kDa), and dextran conjugated with fluorescein (dextran-FITC, *ca.* 150 kDa).

**Figure 6:**
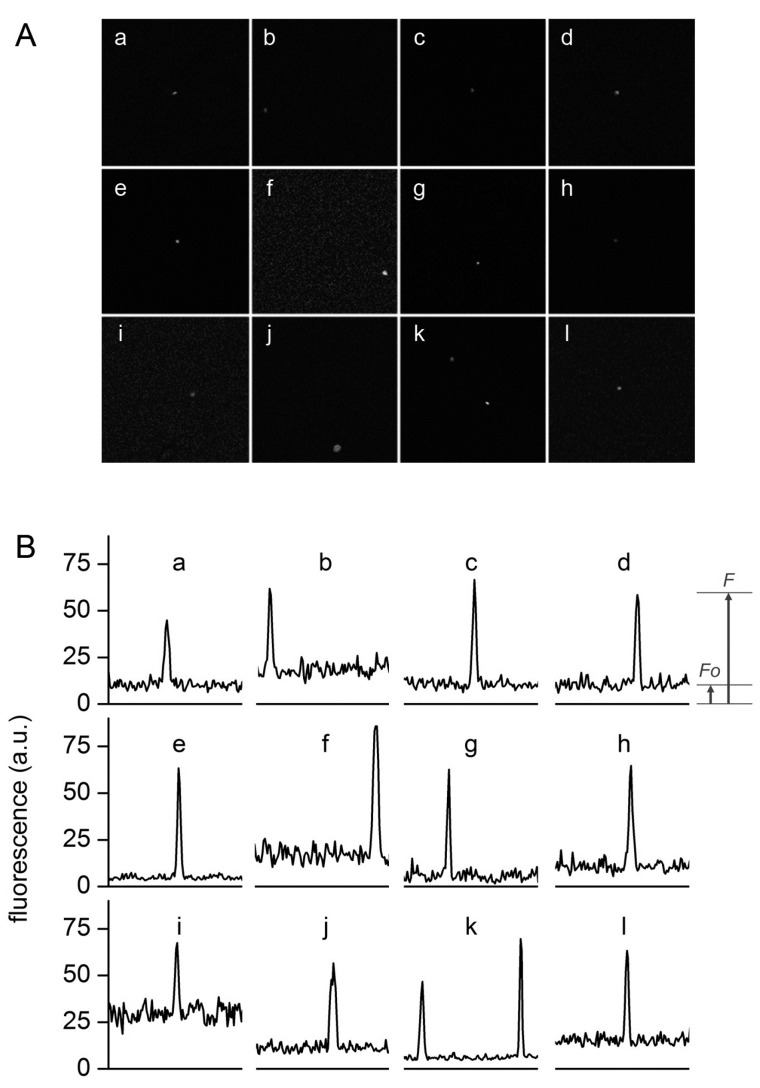
Typical confocal images (**A**) and fluorescence profiles; (**B**) of lipid vesicles whose fluorescence exceeds the fluorescence of the external solution. Solutes: Dextran-FITC (a, b, i), BSA-FITC (c, d, f, g, j, k), dextran-RITC (e), APC (l), PE (h). Methods: M1 (b, e, g, h, i-l), M2 (c, d), M3 (a). The diameters (μm) and the *r*_F_ values of the vesicles are, respectively: (a) 1.3, *4.3*; (b) 1.2, *3.4*; (c) 1.8, *6.1*, (d) 1.5, *6.4*; (e) 1.4, *13*; (f) 1.3, *6.1*; (g) 1.2, *10.6*; (h) 1.3, *6.3*; (i) 2.7, *2.3*; (j) 1.0, *5.5*; (k) 1.5, *7.3* and 1.2, *10.8*; (l) 1.4, *4.2*.

**Figure 7:**
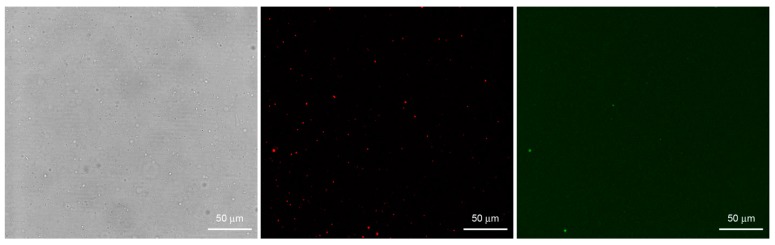
Bright field (**left**) and fluorescence confocal images (**center**, **right**) of lipid vesicles prepared by the film hydration method (M1) starting from a FITC-dextran solution. Vesicles were made of POPC and 0.05 mol% DOPE-lissamine-rhodamine in order to be red-fluorescent (central image). Note that despite the abundant presence of vesicles, only few of them have encapsulated a high amount of green-fluorescent solute (right image) so to appear more fluorescent than the background.

Figure 6 shows a set of typical images obtained by confocal microscopy. Similar images have been obtained for all macromolecular solutes investigated in this work. The results can be summarized as follows. When solutes alone were imaged, a homogeneous fluorescence was always observed. Aggregates were observed only in very rare cases. When vesicles were prepared in the presence of solutes, in all methods and for all lipid concentrations, and for all solutes, we have always observed a small but significant number of vesicles (somehow visible also in bright field) whose fluorescence was higher than the background. If we call *F*_0_ the background fluorescence, and *F* the vesicle fluorescence, it is possible to define, for each visible vesicle, an in/out fluorescence ratio *r_F_* = *F*/*F*_0_ that indicates how many times intra-vesicle fluorescence is higher than background fluorescence, and therefore how many times solutes have been concentrated inside a certain vesicle. As evidenced by carrying out control experiments with fluorescent-labeled lipids (Figure 7), the great majority of vesicles are not visible because their internal fluorescence is very near *F*_0_ (indicating that they have encapsulated the expected number of solutes). On average, less than 1%–2% of vesicles were more fluorescent than the background.

The diameter *d* (μm) of the fluorescent vesicles can be estimated by simply considering that the circular (or quasi-circular) area imaged by confocal scanning is a proxy for the vesicle great circle. Being the optical thickness *ca.* 0.9 μm in our experimental setup, and comparing the vesicle size as appears in bright field images, this approximation appears a viable one.

We have summarized the entrapment results in Table 1. Actually, each experiment was run several times by changing details of the experimental method (for example: lipid concentration, solute concentration, buffer). However, data have been pooled together because despite the variations of the experimental conditions, the differences between the *r_F_* values of each dataset were not statistically significant (*p* > 0.05).

A detailed analysis of the experimental data clearly reveals that there is no correlation between the factors *r*_F_ and the vesicle size, whereas a conclusion on the possible correlation between *r*_F_ and bulk solute concentration remains elusive in this study (statistical analysis provides inconsistent results).

On average, the ˂*r_F_*˃ values lie around 3–4, with some exceptions (5.7, 6.1, 12). This was not unexpected because of the very stochastic nature characterizing the process under study. This behavior (occasional occurrence of high in/out ratios) has also been found in kinetic experiments (see below) as well as in previous studies [9,10,11].

**Figure 8:**
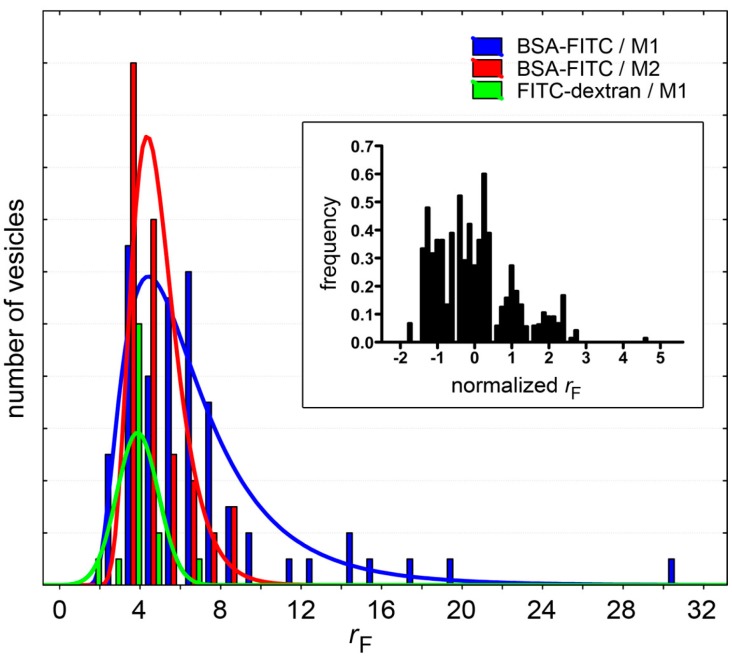
Fluorescence distributions of solute-containing vesicles (only cases with *n* > 48 have been shown, see Table 1). Note that whereas for BSA-FITC the distributions can be fitted with a log-normal curve (red and blue sets), FITC-dextran-containing vesicles have an approximately normal *r*_F_ distribution (green). Inset: To compare different populations (all solutes in Table 1), the distributions of normalized *r*_F_ values are shown; where *r*_F,normalized_ = (*r*_F_ − ˂*r*_F_˃)/SD. It should be noted, however, that since these distributions have been built with a small number of events, their interpretation should be done with caution.

In all cases, the fluorescence distribution is asymmetric (with positive skewness values up to about 2.8), as summarized in Figure 8. This means that there are, on the right-hand side of these distributions, *r*_F_ values that are higher than the average. These maxima (*r_F_*_,max_) are also reported in Table 1, along with the mean *r*_F_ values. These values refer to rare vesicles whose fluorescence is rather high when compared not only to the fluorescence background, but also to the other vesicles. In other words, these vesicles lie at the extreme of the vesicle fluorescence distribution. Note that these very bright vesicles (with *r_F_* > ˂*r_F_*˃) represent a fraction of 0.01%–0.02% of the entire vesicle population.

**Table 1:** Average and maximal *r_F_* values for different solutes and vesicle preparation methods.

Solute	MW (kDa)	Lipids	Method ^c^	˂*r_F_*˃ ± SD (*n*) ^d^	*r_F_*_,max_	Note
Calcein	0.67	POPC	M1	n.a.	n.a.	– ^e^
Dextran-RITC	10	POPC	M1	12 ± 3.8 (11)	19.8	– ^f^
BSA-FITC	66	POPC	M1	5.7 ± 4.5 (69)	29	– ^f^
BSA-FITC	66	PB33PEO29 ^a^	M1	2.9 ± 0.2 (19)	3.2	– ^g^
BSA-FITC	66	POPC	M2	3.7 ± 1.4 (48)	7.7	– ^g^
BSA-FITC	66	POPC ± oleate ^b^	M3	3.5 ± 1.1 (13)	8.6	– ^f^
APC	104	POPC	M1	3.9 ± 1.6 (19)	7.3	– ^h^
Dextran-FITC	150	POPC	M1	2.7 ± 1.0 (15)	5.1	– ^f^
Dextran-FITC	150	POPC ± oleate ^b^	M3	3.8 ± 1.1 (55)	6.4	– ^f^
PE	240	POPC	M1	6.1 ± 2.3 (11)	9.9	– ^i^

^a^ Copolymer vesicle made by Poly(butadiene-b-ethylene oxide), average MW 1.85 kDa; ^b^ POPC:oleate has been varied as 100:0, 80:20, 50:50 (without observing any statistical difference among the samples); ^c^ M1—hydration of thin lipid films deposited on glass beads; M2—hydration of freeze-dried lipid vesicles; M3—ethanol injection method. ^d^ ˂*r_F_*˃, SD, and *n* stand for, respectively, the mean value, the standard deviation, and the number of observations (number of vesicles). ^e^ No vesicles with fluorescence higher than the background were systematically observed; ^f^ [solute]_bulk_ from 0.63 to 5 μM, intravesicle concentration not dependent from vesicle size and solute concentration; ^g^ [BSA-FITC]_bulk_ = 4 μM, intravesicle concentration not dependent from vesicle size; ^h^ [APC]_bulk_ from 0.5 to 5 μM, intravesicle concentration not dependent from vesicle size, yet dependent from solute concentration in inverse way; ^i^ [PE]_bulk_ from 0.16 to 0.25 μM, intravesicle concentration not dependent from vesicle size and solute concentration; “n.a.” stands for “not available”.

Importantly, in analogous experiments with the low molecular weight solute calcein (0.67 kDa, used at a concentration of 1.25–10 μM) it was not possible to detect vesicles with fluorescence higher than the background.

### 3.3. Entrapment of Enzymes—Rate Enhancement

Having shown that the concentration of macromolecules spontaneously entrapped inside some liposomes can be higher than the external concentration by a factor *r_F_*, the next step is to show how this phenomenon impacts on chemical reactions inside liposomes.

Clearly, in those liposomes where the concentrations of chemicals are higher than in the environment, a faster reaction rate is expected. This scenario can be experimentally tested with simple enzyme reactions (we have already seen that in the case of complex, multi-step reactions, such as the protein synthesis, the spontaneous concentration of the macromolecular TX-TL machinery can even trigger the otherwise undetectable or not-occurring protein synthesis).

Here we have developed two simple scenarios: (*i*) an enzyme is encapsulated within vesicles, and a permeable, low-MW substrate is added afterward; (*ii*) an enzyme and a macromolecular substrate are co-encapsulated within vesicles, and reaction occurs immediately.

The data shown in Section 3.2 suggest that the super-concentration effect described in this paper is evident especially for macromolecules. Enzymes are macromolecules and therefore it is expected that when a population of vesicles is formed in an enzyme solution, some of them (usually <1%) will contain a number of enzyme molecules higher than expected. For vesicles of 1–2 μm in diameter, concentration factors around 2.7–6.1 are typically obtained for a variety of solutes (proteins, dextrans). Such values can be used as an educated guess for estimating the behavior of a generic protein that does not establish strong and specific interaction with lipids.

**Figure 9:**
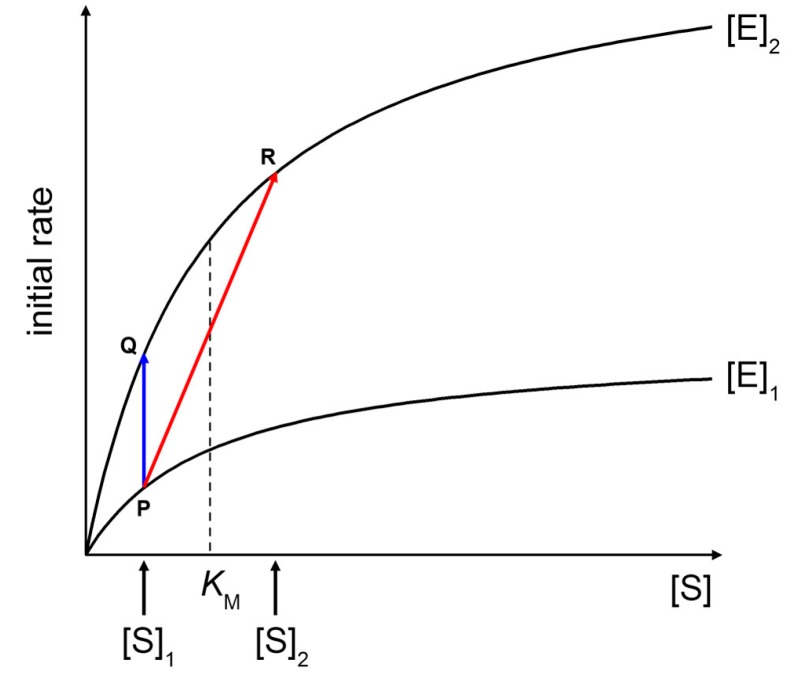
Effects of increasing enzyme and substrate concentration on initial rates of Michaelis-Menten kinetics. See text for details.

Figure 9 shows two Michaelis-Menten plots referring to a hypothetical case of an enzyme that is concentrated three times when encapsulated within vesicles ([E]_2_/[E]_1_ = 3). At a fixed substrate concentration [S]_1_ (in this example, [S]_1_ = *K*_M_/2), an initial rate enhancement of a factor 3 is expected (Figure 9, blue line from P to Q). Note that such a factor is actually independent from the choice of [S]_1_). This example represents the case of macromolecular enzyme and low MW solute, discarding for the moment the problems of substrate permeability rate across the membrane (for a treatment, see below).

If the substrate is also a macromolecule, its encapsulation occurs simultaneously with the enzyme. It can happen that both macromolecules (the enzyme and the substrate) are simultaneously concentrated each by a factor three inside some liposomes ([E]_2_/[E]_1_ = 3, [S]_2_/[S]_1_ = 3). As shown in Figure 9 (red line from P to R), the initial rate enhancement would be of a factor 5.4. Note that in this example [S]_1_ = *K*_M_/2 and [S]_2_ = 3*K*_M_/2, *i.e.*, the substrate concentration moves from below to above *K*_M_. The effect here is not so dramatic because we have supposed a low concentration factor (3×), but if a similar mechanism would increase the substrate concentration by a factor 10 or so—as it happens in conventional sub-micrometer vesicles [9]—one could observe a quite significant rate enhancement inside (few) vesicles.

Here we have studied two experimental systems that recapitulate the two cases illustrated above. In particular, case (*i*) has been modeled with the reaction between carbonic anhydrase and carboxyfluorescein diacetate, whereas for case (*ii*) proteinase K and BSA-FITC have been employed.

#### 3.3.1. Carbonic Anhydrase (CA) and Carboxyfluorescein Diacetate (CFDA)

It is well known that carbonic anhydrase (CA) catalyzes other reactions besides the reversible hydration of CO_2_, in particular, the hydrolysis of esters has been reported [43], as the case of *p*-nitrophenyl acetate [44]. We have employed the membrane-permeable substrate 6-carboxyfluorescein diacetate (CFDA) to carry out an enzymatic reaction inside CA-containing liposomes. Notably, the product of the reaction (6-carboxyfluorescein, CF) is membrane impermeable.

**Figure 10:**
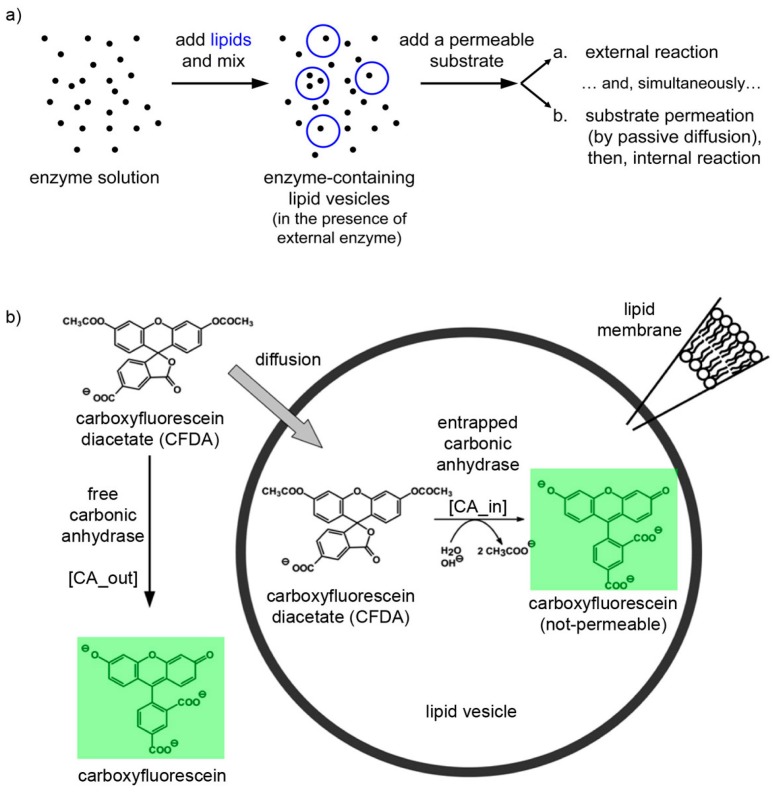
(**a**) Experimental strategy for co-encapsulating an enzyme (carbonic anhydrase, CA, black spots) inside lipid vesicles, followed by adding a membrane-permeable substrate (6-carboxyfluorescein diacetate, CFDA). Note that the external enzyme molecules have not been removed and can react outside vesicles. (**b**) Details of the system under study, showing that CFDA can either react outside vesicles with free CA either permeate through the lipid membrane, reach the vesicle lumen, and then react with the encapsulated CA. The green-fluorescent product, 6-carboxyfluorescein, has been marked by a green box.

The experimental plan is as it follows. Liposomes are formed in a solution containing CA, so that some of them will presumably contain an excess number of CA molecules due to spontaneous super-concentration of this macromolecule (29 kDa) in the liposome lumen. Next, CFDA is added and it will react both with free (external) and encapsulated CA, with the caveats that in the latter case CFDA shall first permeate the liposome membrane (Figure 10). Consequently, a comparison between the rates of bulk and liposomal reaction will reveal whether and what extent super-filled CA-containing liposomes form spontaneously.

**Figure 11:**
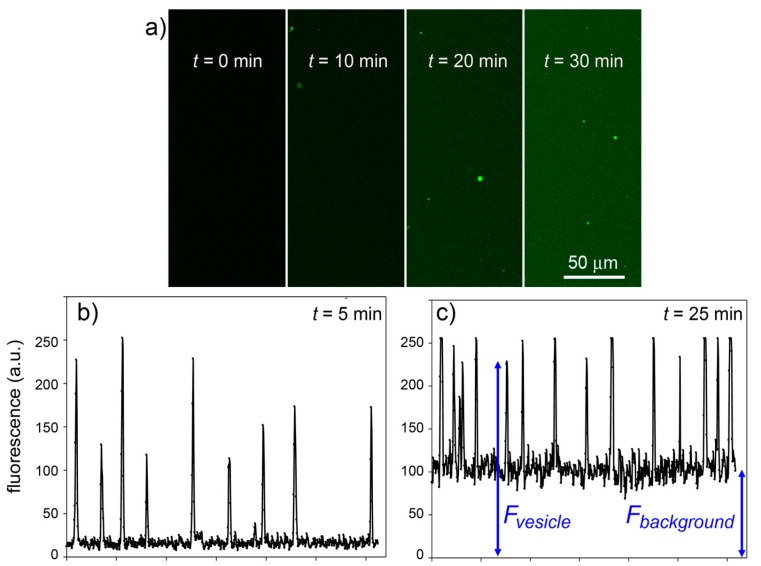
Experimental results for the CA + CFDA system. (**a**) Confocal micrographs showing green fluorescent vesicles that appear in the progress of reaction (*t* = 0, 10, 20, 30 min). The vesicles appear more fluorescent than the background although the same reaction is also occurring outside vesicles. (**b**,**c**) The vesicle fluorescence can be estimated by image analysis (by ImageJ software [45])—here shown as the pixel luminosity of samples taken at 5 and 25 min after mixing. Note that the fluorescence values of each vesicle greatly differ from each other (giving a fluorescence distribution, *cf.* Figure 8) and are well above the background. Note also that the background fluorescence increases with time, as expected.

**Figure 12:**
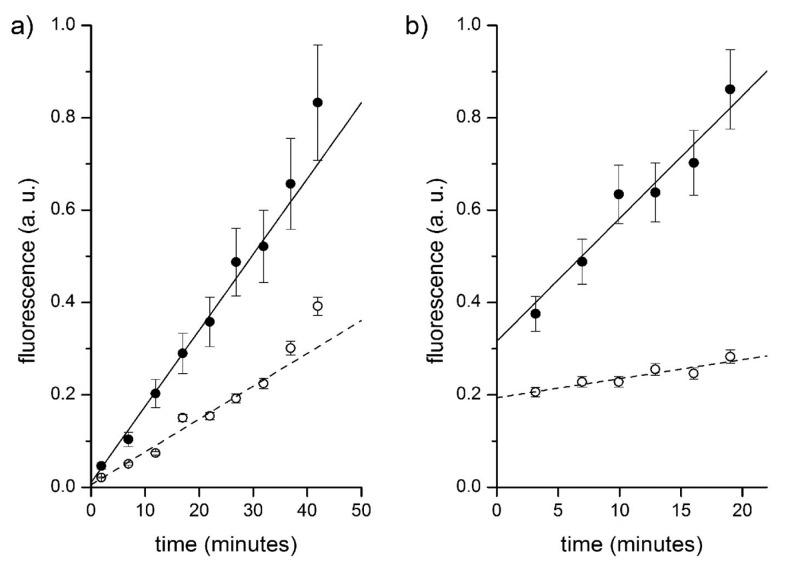
Fluorescence *versus* time profiles of enzymatic reactions inside vesicles (filled circles) and in bulk (empty circles). (**a**) CA-containing vesicles plus externally added CFDA. Data have been fitted by straight lines; the ratio between slopes being 2.3. (**b**) Proteinase K and BSA-FITC containing vesicles. Data have been fitted by straight lines; the ratio between slopes being 6.5.

Experimental results are shown in Figure 11. It is possible to follow the time course of CFDA hydrolysis by confocal microscopy, recording the fluorescence increase at different times, both in bulk than inside liposomes. Figure 11a shows typical microscopic images referring to 0, 10, 20 and 30 min after CFDA addition to CA-containing liposomes (note that free, non-entrapped CA was not removed, so that the fluorescence background also increases in time). By measuring the fluorescence of the liposomes and of the background (Figure 11b,c), at each sampled time, it is possible to plot the rate of enzyme reaction in bulk and inside liposomes. Note that because the liposomes freely float in the solution, it was not possible to follow their individual behavior (namely, the fluorescence increase inside a certain liposome). We therefore captured images containing a number of liposomes and average their fluorescence (the average value, however, is affected by a bias because it is difficult to spot out liposomes that are only slightly more fluorescent than the background; this means that most of the pictures generally contain the brightest liposomes of the population). The fluorescence-*versus*-time profiles (inside liposomes and in bulk) are shown in Figure 12a.

All experiments gave qualitatively similar results, although the numerical reproducibility is hindered by the stochastic nature of the events we are focusing on. Summarizing:

- free (non-encapsulated) CA catalyses the hydrolysis of CFDA, and therefore the background fluorescence increases with a specific rate (*V*_bulk_, in fluorescence units/second) corresponding to the bulk reaction; and
- few liposomes randomly appeared in the illuminated field as bright spots indicating that inside those liposomes CFDA permeated inside the aqueous lumen and reacted with encapsulated *CA.* Being brighter than the background, the amount of CF produced per unit of volume (inside liposomes) is higher than the corresponding quantity in bulk. This intraliposome reaction rate is indicated as *V*_liposome_ (fluorescence units/second).

The ratio *r* between these rates (*r_V_* = *V*_liposomes_/*V*_bulk_) mirrors, at first approximation, the ratio between the CA concentration inside liposomes and in bulk ([CA]_liposome_/[CA]_bulk_), (but see below for a more detailed treatment). It is then possible to estimate the overconcentration of CA inside liposomes in the moment of their formation by measuring the ratio *r_V_*.

As reported in a previous study [14], it is possible to estimate the fraction of liposomes that appear brighter than the background by doping the liposome membrane with fluorescent lipids (this is necessary because the micrometer-sized liposomes used in this study are not well visible in bright field, phase contrast, or Nomarski acquisition modes).

Table 2 summarizes the experimental outcomes from a set of runs carried out by using different CA and CFDA concentration as well as different liposome preparation methods. Table 2 reveals a certain variability among the *r_V_* values of different samples, but the pattern is similar to that recorded in the case of single fluorescent solutes (Table 1). In most cases, the ratio *r_V_* between internal and external reaction rate, which is a measure of the ratio between the internal and external CA concentrations, lies in the 2–4 range. In particular cases, higher values are obtained (e.g., 8.7, 9.1, 11.4). This is due to the very stochastic nature of the phenomenon under study. The difficulty of controlling all microscopic variables affecting the encapsulation process (as well as the sampling procedure) results in a great variability among the experimental outcomes, which had, strictly speaking, a poor reproducibility (in rigorous quantitative sense). Qualitatively, however, all experiments clearly show a common pattern.

**Table 2:** Reaction between encapsulated CA and externally added CFDA.

[CA], μM	[CFDA], μM	Lipids	Method	*r_V_*	Note
0.5	40	POPC	M1	11.4	– ^a^
1.0	40	POPC	M2	2.0	– ^b^
1.0	80	POPC	M2	3.6	– ^c^
0.5	40	POPC/POPG 4/1	M1	2.1	– ^d^
0.5	80	POPC/POPG 4/1	M1	2.3	– ^d^
0.5	160	POPC/POPG 4/1	M1	1.9	– ^d^
0.25	80	POPC/POPG 4/1	M1	8.7	– ^d^
1.0	80	POPC/POPG 4/1	M1	1.3	– ^d^
1.0	40	POPC/oleate 95/5	M3	2.9	– ^e^
0.25	40	POPC/oleate 4/1	M3	9.1	– ^f^
0.5	40	POPC/oleate 4/1	M1	2.5	– ^d^

^a^ [POPC] = 0.5 mM; ^b^ [POPC] = 5 mM, pre-extruded with membranes having pores of 800 nm in diameter; ^c^ [POPC] = 2.5 mM, pre-extruded with membranes having pores of 800 nm in diameter; ^d^ 200 mM HEPES (pH 7) was used as buffer, in combination with lipid film hydration without glass beads, and [POPC] = 0.5 mM; ^e^ 5 mol% oleate helps not to forming too small vesicles; ^f^ 400 mM sodium phosphate (pH 7) was used as buffer.

#### 3.3.2. Numerical Simulations of the CA/CFDA System

In preliminary experiments, we have determined the apparent *K*_M_ and *k*_cat_ values for the reaction, being, respectively, 4.0 ± 0.3 mM and 3.0 ± 0.2 s^−1^. The permeability coefficient of CFDA across the phosphatidylcholine liposomal membrane, namely 10^−7^ cm/s, has been taken from the literature [46]. It is therefore possible to simulate numerically the outcome of experiments as described in Section 3.3.1, namely, the addition of CFDA to CA-containing vesicles, in the presence of non-encapsulated CA.

The system was modeled by dividing the macroscopic sample volume in small “unitary” volumes of *ca.* 50 fL, each containing one liposome (*d* = 1.3 μm; liposome volume = 1.15 fL). These values have been estimated on the basis of the real experimental system. The starting point is a population of CA-containing liposomes suspended in a medium that still contains non-entrapped CA (0.5 μM). Since the enzyme CA is present both inside and outside the liposomes, it is convenient to analyze the system by using the ratio *r*_CA_ = [CA]_in_/[CA]_out_, which can be easily varied, in order to explore three regimes: (*i*) *r*_CA_ < 1 when CA encapsulation is poor; (*ii*) *r*_CA_ = 1 when CA is equally present inside and outside vesicles; (*iii*) and *r*_CA_ > 1 when CA is over-concentrated inside liposomes. CFDA (80 μM), present externally, can react with the external CA according to Michaelis-Menten kinetics, so that the fluorescent carboxyfluorescein (CF) is produced in bulk. In a competitive process, CFDA penetrate inside liposomes through the membrane, and then react with internalized CA. We assume that CF cannot escape from the liposome (the permeability of carboxyfluorescein is negligible for the purposes of this model [47]).

**Figure 13:**
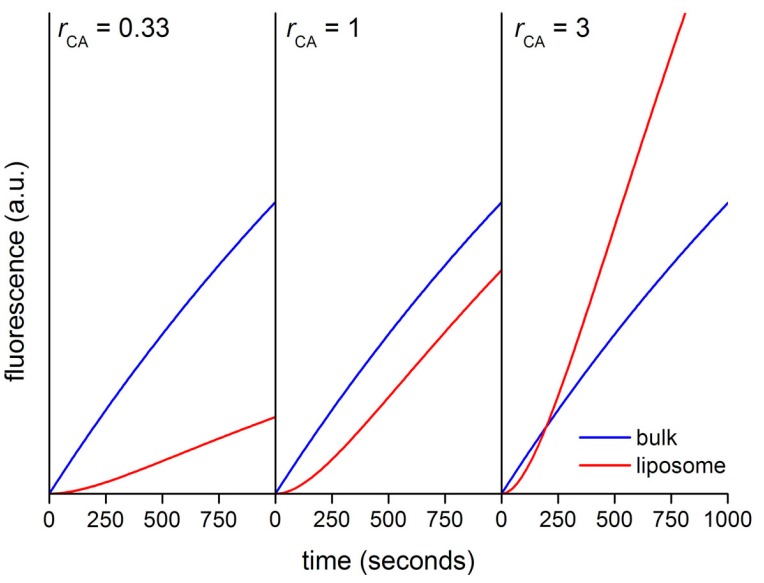
Calculated kinetic profiles for the enzymatic production of carboxyfluorescein from CFDA, under CA catalysis, inside and outside *in silico* vesicle with diameter of 1.3 μm.

Figure 13 shows the calculated kinetic profiles (30 min) for the production of CF in liposomes having *r*_CA_ = 0.33, 1, or 3. When *r*_CA_ = 0.33, the increment of CF concentration, and therefore of its fluorescence is faster in bulk than inside liposomes. The calculated ratio *r*_V_ between the slopes (*V*_in_/*V*_out_) in the quasi-linear region is ~0.28, mirroring in good way the model’s *r*_CA_ value (0.33). When *r*_CA_ = 1 the CF concentration profiles differ only for the presence of a lag phase, due to the retardation effect exerted by the membrane. After the lag phase, in the quasi-linear region, the calculated ratio *r*_V_ ~ 0.82. Finally, when *r*_CA_ = 3 the increase of CF concentration is faster inside liposomes (after a lag phase). According to the model, CA-rich liposomes should soon appear more fluorescent than the background because of a faster accumulation of CF, despite the retardation effect due to the fact that CFDA must cross the membrane before reacting with encapsulated CA. In this case, the calculated *r*_V_ value is ~2.1, which is a proxy value for the true *r*_CA_ value (*r*_CA_ = 3) [48].

#### 3.3.3. Proteinase K and Bovine Serum Albumine-FITC (BSA-FITC)

Fluorescein isothiocyanate-labeled proteins have been used as substrates for proteases [49]; for instance BSA-FITC can be used for this purpose [50]. The commercial BSA-FITC used in this study carries an average of 9.5 FITC moieties, and has relatively low background fluorescence due to autoquenching. Proteolytic digestion alleviates auto-quenching and therefore brings about a concomitant fluorescence increase. This phenomenon provides the basis for a proteolytic assay (with proteinase K) whereby the increase in fluorescence is proportional to the degree of BSA-FITC degradation.

Firstly, we characterized and validated the reaction between proteinase K and BSA-FITC. By comparing the emission spectra of BSA-FITC before and after proteinase K treatment (18 h, 25 °C, pH 7), a fluorescence increase of about 13 times is observed, roughly corresponding to the dequenching of about 3 out of the 7–12 FITC moieties bound to BSA-FITC. It is therefore possible to follow the course of the reaction quite accurately by confocal fluorescence microscopy. On the basis of kinetic analysis, and by using an apparent *K*_m_ ~ 2.3 mM (calculated by us from [50], and further confirmed by other studies based on synthetic peptides as substrates, see [51,52]), we then estimated an apparent *k*_cat_ value of about 1 s^−1^.

**Figure 14:**
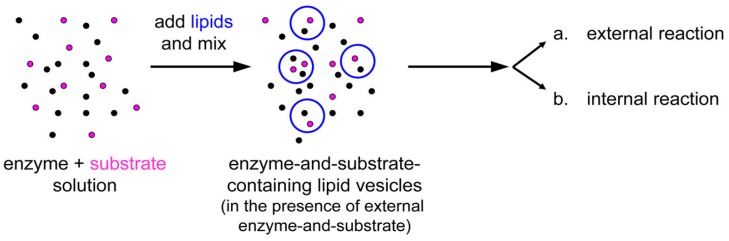
Experimental strategy for co-encapsulating an enzyme (proteinase K, black spots) and a macromolecular substrate (BSA-FITC, pink spots) inside lipid vesicles. Note that the external solutes have not been removed and can react outside vesicles.

Next, we carried out the co-encapsulation experiment, consisting in preparing a mixture of proteinase K (1 μM) and BSA-FITC (2.5–5.0 μM), and forming liposomes *in situ* (Figure 14). The idea is that because both reactants are macromolecules, both can spontaneously concentrate inside liposomes during the membrane closure. For practical reasons (limit of detection), the concentration of BSA-FITC (the substrate) cannot be reduced too much, so that the actual experimental setup does not strictly correspond to the example illustrated in Figure 9 (self-concentration of the substrate from a value below to one above *K*_M_), but it just follows the same principles.

Figure 12b and Table 3 summarize the typical results of the BSA-FITC/proteinase K system. Note that only few vesicles could be detected. Their mean fluorescence *versus* time profile has been reported and fitted with a straight line. The background fluorescence increase has also been recorded. The ratio between these two trends is around 6.5 (first entry of Table 3). Note that the intercepts of these two lines, in this and in other experiments, not always converge to the same value. This could be due to the fact that the sampled vesicles actually contain a higher internal BSA-FITC concentration, which further accelerates the internal reaction. Accordingly, the ratio between the intercepts (in Figure 12b, such ratio is 1.6) is a measure of the ratio between the internal and external BSA-FITC concentration in those vesicles that have been sampled.

**Table 3:** Reaction between co-encapsulated proteinase K and BSA-FITC.

[proteinase K], μM	[BSA-FITC], μM	Lipids	Method	*r*_V_	Note
1	2.5	POPC	M1	6.5	– ^a^
1	5	POPC	M1	2.2	– ^a^
1	2.5	POPC	M2	11.6	– ^b^
1	5	POPC	M2	7.3	– ^b^

^a^ [POPC] = 0.4 mM; ^b^ freeze-dried vesicles obtained from 800 nm-extruded POPC vesicles, [POPC] = 5 mM.

#### 3.3.4. Statistical Considerations

Our data show that macromolecular solutes can be spontaneously encapsulated inside lipid vesicles at a concentration higher than the expected one. The ratio between the actual and the expected concentration (as measured by the *r*_F_ and *r*_V_ values, see Table 1, Table 2 and Table 3) typically lies in the 2–4 range, although *r* values higher than 10 have been also occasionally recorded.

The question is whether such “small” concentration factors (from 2 to 4) can be explained by invoking the natural stochastic fluctuations of the number of solute molecules that can be encapsulated within a vesicle. At this aim we recall our null hypothesis *H*_0_ (see Section 2.2) saying that the fluctuations around the average number *N*_0_ of solute molecules that are encapsulated within a vesicle of volume *V* are ruled by a Poisson distribution. Can stochastic fluctuations account for having an actual number *N* of encapsulated molecules so that *N* > *N*_0_?

Firstly, we calculate *N*_0_ as usual (*N*_0_ = *N*_A_ *C*_0_ *V*), focusing on the experimental conditions giving the lower *N*_0_ value—and stochastic factors are amplified. If natural fluctuations cannot explain data in these conditions, then even more so they do not in all other cases. When 1 μm (diameter) vesicle forms in a *C*_0_ = 0.1 μM solution (the smallest concentration used in this study), *N*_0_ ~ 32. This value (32) means that the Poisson distribution can be approximated by a Gaussian distribution. Fluctuations theory predicts that the magnitude of the fluctuations (Δ*N*) goes as √*N*_0_, *i.e.*, Δ*N* ~ √32 = 5.6. We can then consider a Gaussian distribution of the number of entrapped solute molecules *N*, having average *N*_0_ = 32 and standard deviation Δ*N* = 5.6, and ask what is the probability *p* to find vesicles with *N* > *r* *N*_0_, for *r* ≥ 1. Results are shown in Table 4.

**Table 4:** Entrapment: Gaussian probabilities.

*r*	*r N*_0_	*p*(*N* > *r N*_0_)
1	32	50%
1.41	44.6	1%
2	64	10^−6^%
2.4	76.8	10^−13^%

The Gaussian distribution foresees that the entire vesicle population (99%) should have *r* ≤ 1.41; in other words, local intravesicle concentration factors *r* higher than 1.41 should occur in 1% of cases. The probability of finding vesicles with an internal concentration that is more than two times the expected one (*r* > 2) is instead vanishingly small (*ca.* 10^−6^%), and for the case *r* > 2.4 the value becomes essentially nil (10^−13^%). On the contrary, we have observed vesicles having *r* = 2–4 much more often, *i.e.*, with a frequency of about 0.1%–1%. Even if our estimations of super-filled vesicle abundance were wrong by one, two, or even three orders of magnitude, the difference between the statistical expectations and the observations would be still significant. If this is true in the worst case (*C*_0_ = 0.1 μM), then we can be sure that it is certainly true for higher solutes concentrations (*C*_0_ = 1–10 μM).

In conclusion, this simple statistical analysis confirms that the experimental data presented in this work again represent a conundrum for the physics of solute entrapment, because these “special” vesicles are far more abundant than what is predicted by a random encapsulation. As we suggest in previous work [9,10,11,12,13,14,15], a special mechanism should play a role in generating such intriguing structures.

## 4. Coupled Enzymatic Systems of Interest for Further Investigations

We have demonstrated that simple enzymatic systems can take advantage of micro-compartmentation being concentrated in the aqueous lumen of lipid vesicles. Although this phenomenon occurs in a limited number of vesicles, it is quite interesting because it allows the onset of efficient cell-like reactive systems starting from a diluted solution. However, model systems composed by a single reaction just provide a basic proof of concept, as it was the case of models based on inert proteins (*i.e.*, ferritin encapsulation experiments [10]). On the other hand, we have already investigated the encapsulation of complex systems like the TX-TL reaction mix, showing that the super-concentration phenomenon determines the success of otherwise difficult process, like the synthesis of proteins starting from diluted solutions. Contrary to the simplest cases, the co-encapsulation of several dozens macromolecules challenges the investigation of encapsulation statistics, hampering the construction of accurate theoretical models. In the spectrum of possible case studies, *reactive* systems of intermediate complexity can be advantageous here because they would allow a more precise analysis of co-encapsulation and at the same time function as realistic models of primitive reactive compartments.

Coupled multi-enzymatic systems can be a practical way for proceeding in the above direction. We have already designed some systems—whose reaction progress can be followed by fluorescence microscopy—that readily extend the results presented in this paper, and that are currently under investigation in our laboratory.

**Figure 15:**
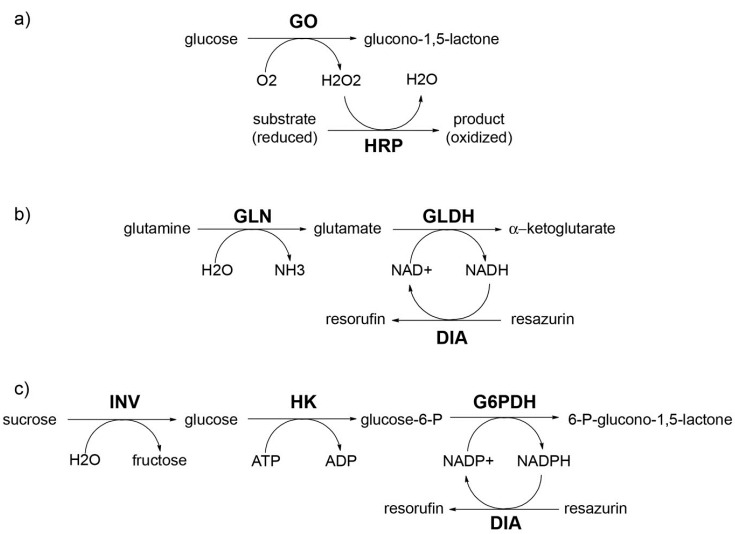
Examples of mini-enzymatic pathways (2, 3, and 4 steps, respectively) that could be constructed inside lipid vesicles. Abbreviations: GO: glucose oxidase; HRP: horseradish peroxidase; GLN: glutaminase; GLDH: glutamate dehydrogenase; DIA: diaphorase; INV: invertase; HK: hexokinase; G6PDH: glucose-6-phosphate dehydrogenase.

The strategy is based on the production of a fluorescent molecule downstream of a multi-step enzymatic pathway. In this respect, two enzymes are particularly useful, *i.e.*, peroxidase and diaphorase (*i.e.*, a NAD(P)H dehydrogenase). Peroxidase uses hydrogen peroxide for oxidizing fluorogenic substrates such as reduced fluorescein (fluorescin), reduced rhodamine, or the so-called Amplex Red^©^ (a phenoxazine derivative) to give, respectively, fluorescein, rhodamine, and resorufin. Diaphorase uses NAD(P)H for reducing resazurin to resorufin. The activity of these two enzymes can be combined with other reactions in order to build fluorescence-detectable enzyme-catalyzed mini-pathways to be reconstructed inside vesicles. Figure 15 shows just some examples of such possible systems.

Clearly, several practical issues must be assessed for finding the best model system that can be effectively used in vesicle systems, first of all the permeability issue for all substrates, the distance from optimal environmental conditions (e.g., pH) for each enzyme, and the possibility of inferring the local enzyme concentration from setting proper experimental parameters and from observables. Numerical simulations can be helpful both for design and analysis.

## 5. Concluding Remarks

In this paper we have firstly reviewed the previous results on protein and ribosome encapsulation inside vesicles, then presented new data. In contrast to previous studies, which have been carried out on conventional vesicles visualized by cryo-TEM, the present paper deals with 1–2 μm-sized vesicles and confocal fluorescence microscopy analysis. We have reported that macromolecules like proteins and dextrans can be over-encapsulated in micrometer-sized vesicles reaching a local intravesicle concentration of about 2–4 times higher than the expected value (and occasionally up to 10 times higher). Even if these enhancement factors appear to be low, they overcome the theoretical expectations, based on the Poisson (or Gaussian) distribution. As happened in previous study, the over-concentration phenomenon concerns only a small vesicle subpopulation (<1%). Moreover, in this study we have also included “dynamic” system, e.g., enzymatic systems, to show how the spontaneous over-concentration of solutes inside vesicles implies an enhanced reactivity of intra-vesicle milieu when compared with the external environment.

In addition to the obvious general—and not yet properly emphasized—message that vesicle populations are heterogeneous in terms of solute content (for a discussion, see also [53]), the main conclusion from this study is that particular physico-chemical conditions operate in a way to promote the formation of vesicles super-filled of solutes (where, by “super-filled”, we mean filled with a number of solute that exceed the expected number also keeping into account the stochastic fluctuations).

We maintain that such phenomenology might have had a role in promoting the origin of early functional cells, by accumulating bioactive materials inside vesicle lumen so that reactions in confined microenvironment could occur much better than in the external (bulk) phase. Moreover, this view emphasize the active role of lipid compartments in favoring the onset of metabolism, in the sense that lipid compartments, which form spontaneously in solution, not only provide a protected microenvironment for early reactions, and at the same time equip the compartment with a semi-permeable membrane, but also concur to the encapsulation of solutes and therefore to the very mechanism of protocell formation from separated components. Despite the fact that the super-filled vesicles represent only a small fraction of the entire population, their absolute number is nevertheless remarkable (e.g., 0.1% of a “diluted” vesicle sample, e.g., [lipids] = 1 μM, consists of *ca.* 10^5^ micrometer-sized vesicle/mL).

Among the possible open questions and future directions related to this research, in addition to the investigation on the generative mechanism—also related to the power-law profiles [10,11]—the issue of nucleic acid entrapment plays a central role, especially if non-phospholipids compartments are considered, like fatty acids vesicles. Our preliminary experiments are very promising (D’Aguanno *et al.*, manuscript in preparation). It is worth mentioning that not only vesicles composed by pure compounds can be used (e.g., pure oleate vesicles), but it would be interesting to explore the behavior of lipid mixtures. A realistic scenario for primitive membranes indeed includes mixture of simple amphiphiles, with different chain length and head groups. As revealed by recent studies, such membranes might show intriguing features [54,55,56], but the issue of solute encapsulation has not been faced yet. Another interesting direction deals with the abovementioned reconstruction of multi-step pathways (model systems shown in Figure 15), and of exploiting the super-concentration effect to assemble high-order supramolecular structures inside lipid compartments.

## 6. Experimental Section

*Materials*. Commercially available products have been used, in particular, (*a*) from Sigma-Aldrich: dextran conjugated with rhodamine (dextran-RITC, #R8881, MW *ca.* 10 kDa), *R*-phycoerythrin (PE, #52412, MW *ca.* 240 kDa), bovine serum albumine conjugated with fluorescein (BSA-FITC, #A9771, MW *ca.* 66 kDa), allophycocyanin (APC, #A7472, MW *ca.* 104 kDa), dextran conjugated with fluorescein (dextran-FITC, #FD-150S, MW *ca.* 150 kDa), carbonic anhydrase (CA, #C3934, MW *ca.* 29 kDa), 6-carboxyfluorescein diacetate (CFDA, #C5041, 460 Da), 6-carboxyfluorescein (6-CF, #C0662, 376 Da), calceina disodium salt (#21030, 666 Da), oleic acid (#O1008), sodium oleate (#O7501), and all solvents and buffers; (*b*) from Invitrogen: proteinase K (MW *ca.* 40 kDa); (*c*) from Avanti Polar Lipids: 1-palmitoyl-2-oleoyl-sn-glycero-3-phosphatidylcholine (POPC), 1-palmitoyl-2-oleoyl-sn-glycero-3-phosphatidylglycerol (POPG) 1,2-dioleoyl-sn-glycero-3-phosphatidyl ethanolamine-lissamine-rhodamine (DOPE-lissamine-rhodamine). The block copolymer poly(butadiene-b-ethylene oxide) (PB33PEO29), MW *ca.* 1.85 kDa, was a generous gift from Prof. Stephan Förster (Bayreuth University, Germany).

*Methods*. Lipid vesicles were prepared by three methods: film hydration method (M1); rehydration of pre-formed extruded and freeze-dried vesicles (M2); and ethanol injection method (M3). Details of the three methods can be found in the Supplementary File of reference [14]; here we summarize only the essential points.

Film hydration method (M1). Lipid-covered glass beads (diam. 2 mm) were prepared by the evaporation method. In particular, the lipid mixture (POPC plus other lipids such as POPG or oleic acid or DOPE-lissamine-rhodamine) was dissolved in chloroform and placed in a round-bottomed flask. Glass beads (measured by weight) were added and the solvent was removed at reduced pressure. Lipid-covered glass beads were further dried at high vacuum. The average amount of lipid/bead, measured by the Stewart assay (see [14] for details), was 10 ± 1 nmol_lipids_/bead. Vesicles were obtained by hydrating the so-prepared beads with a solute-containing aqueous buffer. For example, 10 beads (100 nmol_lipids_) were hydrated with 200 μL solution to obtain vesicles with a lipid concentration of 0.5 mM. Care should be taken to re-suspend effectively the lipids by continuous pipetting up and down the aqueous solution. Note that essentially similar results have been obtained when the lipid film was directly prepared in a Eppendorf-like tube, starting from lipid solution in methanol, and next evaporation of the solvent by a centrifugal evaporator (e.g., Savant SpeedVac^®^ or similar).

Rehydration of extruded and freeze-dried vesicles (M2). POPC vesicles were first prepared by the thin film hydration method. Vesicles were extruded by 10 passages over two stacked Whatman Nuclepore Track-Etch polycarbonate membranes mounted on a hand-extruder (Avestin Liposofast) with pore size 400 or 800 nm. The so-obtained extruded vesicles were freeze-dried so to obtain a vesicle “cake” that was then hydrated with an appropriate amount of solute-containing solution. The final POPC concentration was 2.5–5 mM.

Ethanol injection method (M3). An ethanol solution of lipids (100 mM) was injected, by means of a Hamilton microsyringe or a Gilson micropipette, in the solution containing the solute(s) of interest, in a 1:30 to 1:60 volume ratio, so that the final lipid concentration was 1.7–3.3 mM and the final ethanol amount was 1.7%–3.3% v/v. The lipids used were: (*i*) POPC; (*ii*) POPC/sodium oleate 4/1 mol/mol. In the latter case, the stock solution was prepared from POPC/ethanol and sodium oleate/methanol stock solutions.

Preliminary experiments were carried out to select the best buffer. 50–200 mM HEPES (pH 7.4) as well as phosphate-buffered saline (PBS, pH 7.4) produced mostly vesicle aggregates. Phosphate buffers (pH 7) produced vesicle aggregates at high phosphate concentration (200–400 mM), and good vesicle (both for POPC or POPC:oleate 4:1 mol/mol) samples at low phosphate concentration (50–100 mM). Britton-Robinson buffer (pH 7) gave different results depending on the lipids used.

The above mentioned methods have been used to prepare solute-containing vesicles, in particular, the following solution have been used: BSA-FITC (0.63–5 μM), dextran-FITC (0.63–5 μM), dextran-RITC (0.63–5 μM), PE (0.16–0.25 μM), APC (0.5–5 μM), CA (0.25–1 μM), proteinase K plus BSA-FITC (1 μM plus 2.5–5 μM, respectively). The buffer was 100 mM sodium phosphate (pH 7) if not specified otherwise (see Table 1, Table 2 and Table 3 footnotes). The non-encapsulated solutes were never removed.

Carbonic anhydrase (CA) plus carboxyfluorescein diacetate (CFDA) reaction was carried out as it follows. First, CA-containing vesicles were prepared by one of the above-described methods. Next, CFDA was added from a stock acetonitrile solution (the final acetonitrile content was ≤5 vol%). After CFDA addition (<20 s), the sample was quickly transferred in the glass chamber and imaged by confocal microscopy.

Proteinase K plus BSA-FITC reaction was carried out by forming vesicles in a freshly prepared solution which contained proteinase K (1 μM) plus BSA-FITC (2.5–5 μM). After vesicle formation (<1 min), the sample was quickly transferred in the glass chamber and imaged by confocal microscopy.

Glassware preparation for microscopy analysis. All glassware was cleaned with ethanol and lint-free paper (Kimwipes Lite, Kimberly-Clark) to remove dust and contaminants from the surface. The observation chamber was homemade by creating a properly shaped spacer with two parafilm layers placed between the glass slide and the cover slip. This three-layer construction was placed over a heater plate to melt the parafilm. In each “chamber” about 20–40 μL of each solution can be placed.

Confocal microscopy analysis was carried out by using (*i*) Zeiss LSM 510 inverted microscope or (*ii*) Leica TCS SP5 inverted microscope. Laser power, gain, offsets were optimized for each experiment in order not to saturate the detector. This was particularly important in kinetic experiments, where fluorescence increases in time. Calibration lines were first constructed by using increasing amount of solutes (dextran-RITC, dextran-FITC, BSA-FITC, APC, PE, 6-carboxyfluorescein), in order to convert fluorescence units (8-bits values, from 0 to 255) in molar concentration. In no cases we observed self-quenching or non-linear responses, also thanks to the small sample thickness. Preliminary experiments were carried out by recording the fluorescence profile in *z* direction in order to determine the most suitable depth of focal plane (generally occurring at >5 μm from the surface), to avoid surface scattering and other surface-related phenomena. The fluorescence intensity of the vesicles and of the background was quantified by image analysis carried out by ImageJ software (http://rsbweb.nih.gov/ij/) [45].

## References

[1] Ruiz-Mirazo K., Briones C., de la Escosura A. (2014). Prebiotic Systems Chemistry: New Perspectives for the Origins of Life. Chem. Rev..

[2] Luisi P.L. (2006). The Emergence of Life: From Chemical Origins to Synthetic Biology.

[3] Luisi P.L., Ferri F., Stano P. (2006). Approaches to Semi-Synthetic Minimal Cells: A Review. Naturwissenschaften.

[4] Berclaz N., Muller M., Walde P., Luisi P.L. (2001). Growth and Transformation of Vesicles Studied by Ferritin Labeling and Cryotransmission Electron Microscopy. J. Phys. Chem. B.

[5] Berclaz N., Blochliger E., Muller M., Luisi P.L. (2001). Matrix Effect of Vesicle Formation as Investigated by Cryotransmission Electron Microscopy. J. Phys. Chem. B.

[6] Oberholzer T., Nierhaus K.H., Luisi P.L. (1999). Protein Expression in Liposomes. Biochem. Biophys. Res. Commun..

[7] Oberholzer T., Luisi P.L. (2002). The Use of Liposomes for Constructing Cell Models. J. Biol. Phys..

[8] Murtas G., Kuruma Y., Bianchini P., Diaspro A., Luisi P.L. (2007). Protein Synthesis in Liposomes with a Minimal Set of Enzymes. Biochem. Biophys. Res. Commun..

[9] Souza T.P., Stano P., Luisi P.L. (2009). The Minimal Size of Liposome-Based Model Cells Brings about a Remarkably Enhanced Entrapment and Protein Synthesis. Chembiochem Eur. J. Chem. Biol..

[10] Luisi P.L., Allegretti M., de Souza T.P., Steiniger F., Fahr A., Stano P. (2010). Spontaneous Protein Crowding in Liposomes: A New Vista for the Origin of Cellular Metabolism. Chembiochem.

[11] Souza T.P., Steiniger F., Stano P., Fahr A., Luisi P.L. (2011). Spontaneous Crowding of Ribosomes and Proteins inside Vesicles: A Possible Mechanism for the Origin of Cell Metabolism. Chembiochem.

[12] Souza T.P., Stano P., Steiniger F., D’Aguanno E., Altamura E., Fahr A., Luisi P.L. (2012). Encapsulation of Ferritin, Ribosomes, and Ribo-Peptidic Complexes Inside Liposomes: Insights Into the Origin of Metabolism. Orig. Life Evol. Biospheres.

[13] Luisi P.L. (2012). An Open Question on the Origin of Life: The First Forms of Metabolism. Chem. Biodivers..

[14] Stano P., D’Aguanno E., Bolz J., Fahr A., Luisi P.L. (2013). A Remarkable Self-Organization Process as the Origin of Primitive Functional Cells. Angew. Chem. Int. Ed..

[15] De Souza T.P., Fahr A., Luisi P.L., Stano P. (2014). Spontaneous Encapsulation and Concentration of Biological Macromolecules in Liposomes: An Intriguing Phenomenon and Its Relevance in Origins of Life. J. Mol. Evol..

[16] Kruger K., Grabowski P.J., Zaug A.J., Sands J., Gottschling D.E., Cech T.R. (1982). Self-Splicing RNA: Autoexcision and Autocyclization of the Ribosomal RNA Intervening Sequence of Tetrahymena. Cell.

[17] Zhang B.L., Cech T.R. (1998). Peptidyl-Transferase Ribozymes: Trans Reactions, Structural Characterization and Ribosomal RNA-like Features. Chem. Biol..

[18] Joyce G.F. (1996). Building the RNA World. Ribozymes. Curr. Biol. CB.

[19] Szostak J.W., Bartel D.P., Luisi P.L. (2001). Synthesizing Life. Nature.

[20] Mavelli F. (2012). Stochastic Simulations of Minimal Cells: The Ribocell Model. BMC Bioinform..

[21] Stano P., Carrara P., Kuruma Y., de Souza T.P., Luisi P.L. (2011). Compartmentalized Reactions as a Case of Soft-Matter Biotechnology: Synthesis of Proteins and Nucleic Acids inside Lipid Vesicles. J. Mater. Chem..

[22] De Lorenzo V., Danchin A. (2008). Synthetic Biology: Discovering New Worlds and New Words. EMBO Rep..

[23] Chiarabelli C., Stano P., Luisi P.L. (2009). Chemical approaches to synthetic biology. Curr. Opin. Biotech..

[24] Gil R., Silva F.J., Peretó J., Moya A. (2004). Determination of the Core of a Minimal Bacterial Gene Set. Microbiol. Mol. Biol. Rev. MMBR.

[25] Moya A., Gil R., Latorre A., Peretó J., Pilar Garcillán-Barcia M., de la Cruz F. (2009). Toward Minimal Bacterial Cells: Evolution *vs.* Design. FEMS Microbiol. Rev..

[26] Shimizu Y., Inoue A., Tomari Y., Suzuki T., Yokogawa T., Nishikawa K., Ueda T. (2001). Cell-Free Translation Reconstituted with Purified Components. Nat. Biotechnol..

[27] Shimizu Y., Kanamori T., Ueda T. (2005). Protein Synthesis by Pure Translation Systems. Methods.

[28] Lasic D.D. (1988). The Mechanism of Vesicle Formation. Biochem. J..

[29] Van Hoof B., Markvoort A.J., van Santen R.A., Hilbers P.A.J. (2012). On Protein Crowding and Bilayer Bulging in Spontaneous Vesicle Formation. J. Phys. Chem. B.

[30] Van Hoof B., Markvoort A.J., van Santen R.A., Hilbers P.A.J. (2014). Molecular Simulation of Protein Encapsulation in Vesicle Formation. J. Phys. Chem. B.

[31] Walde P. (2006). Surfactant Assemblies and Their Various Possible Roles for the Origin(S) of Life. Orig. Life Evol. Biosph..

[32] Matthäus C., Bird B., Miljković M., Chernenko T., Romeo M., Diem M. (2008). Infrared and Raman Microscopy in Cell Biology. Methods Cell Biol..

[33] Nishimura K., Hosoi T., Sunami T., Toyota T., Fujinami M., Oguma K., Matsuura T., Suzuki H., Yomo T. (2009). Population Analysis of Structural Properties of Giant Liposomes by Flow Cytometry. Langmuir ACS J. Surf. Colloids.

[34] Nishimura K., Matsuura T., Nishimura K., Sunami T., Suzuki H., Yomo T. (2012). Cell-Free Protein Synthesis inside Giant Unilamellar Vesicles Analyzed by Flow Cytometry. Langmuir ACS J. Surf. Colloids.

[35] Dominak L.M., Keating C.D. (2007). Polymer Encapsulation within Giant Lipid Vesicles. Langmuir.

[36] Bangham A.D., Standish M.M., Watkins J.C. (1965). Diffusion of Univalent Ions across the Lamellae of Swollen Phospholipids. J. Mol. Biol..

[37] Nourian Z., Roelofsen W., Danelon C. (2012). Triggered Gene Expression in Fed-Vesicle Microreactors with a Multifunctional Membrane. Angew. Chem. Int. Ed. Engl..

[38] Shew R.L., Deamer D.W. (1985). A Novel Method for Encapsulation of Macromolecules in Liposomes. Biochim. Biophys. Acta.

[39] Ishikawa K., Sato K., Shima Y., Urabe I., Yomo T. (2004). Expression of a Cascading Genetic Network within Liposomes. FEBS Lett..

[40] Batzri S., Korn E.D. (1973). Single Bilayer Liposomes Prepared without Sonication. Biochim. Biophys. Acta BBA—Biomembr..

[41] Domazou A.S., Luisi P.L. (2002). Size Distribution of Spontaneously Formed Liposomes by the Alcohol Injection Method. J. Liposome Res..

[42] Stano P., Bufali S., Pisano C., Bucci F., Barbarino M., Santaniello M., Carminati P., Luisi P.L. (2004). Novel Camptothecin Analogue (gimatecan)-Containing Liposomes Prepared by the Ethanol Injection Method. J. Liposome Res..

[43] Tashian R.E., Douglas D.P., Yu Y.S. (1964). Esterase and hydrase activity of carbonic anhydrase. I. From primate erythrocytes. Biochem. Biophys. Res. Commun..

[44] Verpoorte J.A., Mehta S., Edsall J.T. (1967). Esterase activities of human carbonic anhydrases B and C. J. Biol. Chem..

[45] Schneider C.A., Rasband W.S., Eliceiri K.W. (2012). NIH Image to ImageJ: 25 years of image analysis. Nat. Methods.

[46] Thomae A.V. (2007). Experimental and Theoretical Investigations on Lipid Bilayer Permeation. Ph.D. Thesis.

[47] New R.R.C. (1990). Liposomes. A Practical Approach.

[48] Hoops S., Sahle S., Gauges R., Lee C., Pahle J., Simus N., Singhal M., Xu L., Mendes P., Kummer U. (2006). COPASI—a COmplex PAthway SImulator. Bioinformatics.

[49] De Lumen B.O., Tappel A.L. (1970). Fluorescein-hemoglobin as a substrate for cathepsin D and other proteases. Anal. Biochem..

[50] Voss E.W., Workman C.J., Mummert M.E. (1996). Detection of Protease Activity Using a Fluorescence-Enhancement Globular Substrate. BioTechniques.

[51] Kristjánsson M.M., Magnússon Ó.T., Gudmundsson H.M., Alfredsson G.Á., Matsuzawa H. (1999). Properties of a subtilisin-like proteinase from a psychrotrophic Vibrio species. Comparison with proteinase K and aqualysin I. Eur. J. Biochem..

[52] Georgieva D., Genov N., Voelter W., Betzel C. (2006). Catalytic Efficiencies of Alkaline Proteinases from Microorganisms. Z. Naturforsch C.

[53] Stano P., Souza T., Carrara P., Altamura E., D’Aguanno E., Caputo M., Luisi P.L., Mavelli F. (2015). Recent biophysical issues about the preparation of solute-filled lipid vesicles. Mech. Adv. Mat. Struct..

[54] Mansy S.S., Schrum J.P., Krishnamurthy M., Tobé S., Treco D.A., Szostak J.W. (2008). Template-directed synthesis of a genetic polymer in a model protocell. Nature.

[55] Maurer S.E., Deamer D.W., Boncella J.M., Monnard P.A. (2009). Chemical evolution of amphiphiles: Glycerol monoacyl derivatives stabilize plausible prebiotic membranes. Astrobiology.

[56] Budin I., Prywes N., Zhang N., Szostak J.W. (2014). Chain-length heterogeneity allows for the assembly of fatty acid vesicles in dilute solutions. Biophys. J..

